# Green care in first-episode psychosis: short report of a mixed-methods evaluation of a ‘woodland group’ in an early intervention service

**DOI:** 10.1192/bjb.2021.54

**Published:** 2021-08

**Authors:** Sharon Cuthbert, Harriet Sharp, Clio Berry

**Affiliations:** 1 Sussex Partnership NHS Foundation Trust, Hove, UK; 2 Brighton and Sussex Medical School, Brighton, UK

**Keywords:** Outcome studies, out-patient treatment, psychosocial interventions, qualitative research, psychotic disorders

## Abstract

**Aims and method:**

In the context of increasing recognition of the role of nature in well-being, but limited evidence for specific patient groups, we describe a mixed-methods evaluation of a 10-week green care intervention (a woodland group) for 18- to 30-year-olds who had experienced a first episode of psychosis. Data were collected using the Questionnaire on the Process of Recovery (QPR), semi-structured service evaluation questionnaires, the NHS Friends and Family Test (FFT), and focus group analysis.

**Results:**

All participants present at week 10 (*n* = 5) would recommend this group to others; 4/8 participants showed reliable improvement on QPR outcome measures. Thematic analysis identified themes of connection with nature and others, development of a sense of well-being and ‘peacefulness’ and new perspectives on psychotic experience.

**Clinical implications:**

This small retrospective evaluation describes patient-reported benefits, feasibility and acceptability of green care interventions within early intervention in psychosis services (EIS).

Early intervention services (EIS) aim to reduce symptomatic periods and improve developmental and social trajectories for those who have experienced a first episode of psychosis (FEP).^1^ Full recovery and therapeutic engagement may be difficult to achieve.^2–4^ Green care uses natural environments to facilitate improvements in well-being, with growing evidence to support effectiveness in the general population.^5,6^ Evaluations suggest that people with mental health problems may benefit proportionally more from such nature-based care.^7^ Accessing green space may mitigate health inequalities closely tied to severe and enduring mental illness (SMI),^8,9^ including for young people, among whom nature ‘connectedness’ is low.^10^ Groups in natural spaces may also confer potential advantages for therapeutic engagement and work.^11–13^ However, there is limited evidence for green care for those with SMI^14,15^ and, to our knowledge, none in FEP. Green care is a ‘complex intervention’, combining psychological work, physical activity and social interactions,^16^ so our study used a mixed-methods approach, as recommended by the Medical Research Council.^17^ This mixed-methods evaluation considers potential benefits and barriers encountered in a green care programme delivered in an EIS.

## The intervention

The woodland group was facilitated by Circle of Life Rediscovery (CLR), commissioned by Sussex Partnership NHS Foundation Trust for 10 weekly half-day sessions. Two CLR staff prepared and attended groups, collaborating with EIS staff who facilitated transport and supported individuals (up to 15), with an overall staff:participant ratio of at least 1:3.

The group included a short walk, refreshments, contemplative time and activities such as learning about plants and habitats, maintaining the woodland area and cooking.

All participants were aged between 18–30 and had experienced FEP, some demonstrating active psychotic symptoms. Target problems included isolation, anxiety and depression.

## Ethics and consent

This study was conducted as a service evaluation and did not require research ethics approval. All participants gave written consent for data to be collected and used within evaluations of the service.

## Data collection and analysis

Participants completed the 15-item Questionnaire about the Process of Recovery (QPR) at weeks 1, 3, 6 and 10. The QPR has good internal consistency, high test–retest reliability and convergent validity.^18,19^ ‘Reliable change’, an estimate of statistical significance of change in outcome scores, was calculated using the Jacobson–Truax formula with published Cronbach's alpha and standard deviation.^20,21^ Participants in the final session also completed a semi-structured service evaluation questionnaire and the NHS Friends and Family Test (FFT).^22^

Qualitative data were invited through free-text portions of the evaluation questionnaire and through intervention summaries completed by participants and staff. A 20 min focus group (*n* = 7) was convened in the final session, facilitated by the group leader and transcribed by the first author. All three authors independently performed thematic analysis of the data.

## Results

The QPR 5-point Likert scale ranges from 1 (disagree strongly) to 5 (agree strongly). Mean QPR scores increased from 3.4 (*n* = 3) at week 1 to 3.8 at week 10 (*n* = 8). Four patients showed reliable improvement and one showed reliable deterioration for those with data at two time points (*n* = 8).

All participants present at week 10 (*n* = 5) recommended the group on the FFT.^22^

Results of the evaluation questionnaire (at week 10) are shown in Table 1.

**Table 1:** Participants’ (*n* = 8) scores on the service evaluation questionnaire (at week 10)

	Mean (median)^a^
I feel more connected to the world around me	4.4 (4)
My emotional and psychological well-being has improved	4.3 (4)
My recovery has been helped	4.1 (4)
I have found ways to manage difficult emotions	3.5 (3.5)
I have found things that can help me in a crisis	3.8 (4)
I have connected with other people in a positive way	4.8 (5)
I have had some difficult social encounters	3.4 (3)
I have been more worried about the world around me	2.5 (2.5)
I feel more hopeful	3.9 (4)
I have felt more confident about going to new places	3.4 (4)
I have learnt new skills	4.3 (4)
I have felt more confident about meeting people	4 (4)
I have made friendships which may continue	4.1 (4)

a. Scores are on a 5-point Likert scale: 1, ‘strongly disagree’, 5 ‘strongly agree’.

All authors were concordant on two higher-order themes from the qualitative data. The first was connection with others and nature. Participants described reduced isolation and improved relationships:

‘It's therapeutic to sit round a fire with other people […] it's nice to feel connected.’

‘The difference […] I'm not isolated […] I can feel normal. You go to doctor's appointments, you're not part of the 9–5 but we are still here.’

‘I feel […] awe/curiosity for the natural world.’

A second overarching theme was of positive change in self, including skills development and emotional change. This was expressed as feeling ‘confident to do things’ or ‘I enjoyed the cooking […] it helped distract me more, and it's skills I have used at home’. Participants appreciated creativity and the chance to ‘take something away’ – including physical objects, memories and new skills. They described feelings of calmness: ‘100% impact. I feel better about myself […] I feel supported, I feel able. I have found stillness, calmness […]. It is very healing’. Staff noted that participants appeared more relaxed in the woodland group than in other settings.

Particularly relevant to this group were repeated suggestions that the group enabled changed perspectives on psychosis. One participant wrote ‘[it] showed me what's real in my […] psychotic state’ and another ‘[it] helps me understand other people's perception of psychosis’.

Barriers to participation included short sessions and poor weather. No adverse events, near misses or concerns regarding risky behaviour were reported.

## Discussion and conclusions

This evaluation offers promise for green care as an intervention in FEP. Unusually, group attendance improved over time and all participants recommended it. Sequential QPR measures showed positive trends across all recovery domains; additionally, attendees described increased insight and contextualisation of difficulties, alongside distraction from problematic symptoms. Participants spontaneously discussing their beliefs and experiences, potentially enabled by the attention-restoring and stress-reducing properties of the woodland environment, seems to have encouraged reflection and reality testing. The group was therapeutic, with features of universality, development of socialising skills and interpersonal learning.^23^

Feedback for improvement was sparse. The location, with associated travelling costs, and the intensive EIS staff involvement were cited as barriers to re-commissioning. CLR staff reported that the ‘scaffolding’ provided by NHS support enabled them to facilitate the group. Without this early input, successful engagement of this patient group seems less likely. Full economic (and carbon) costings, including staff resource, should be part of future evaluations and commissioning for such groups, as within the wider social prescribing context.^24^

The findings are limited by small sample size, areas of incomplete data and use of patient-reported outcome scales only. We have no data from those who chose not to attend the intervention. Although qualitative analysis allows themes to emerge from the data, defining components and aspects of the intervention which may be poorly understood,^25^ thematic saturation was likely not reached, and participants may have felt constrained by facilitator presence in the focus group. Further exploration of the increased reflective ability on personal psychotic experiences that participants reported feeling within the grounding and supported woodland setting would be particularly helpful.

Initiatives to boost green prescribing are expanding.^26^ This is a preliminary report, indicating promising features for green care as a sustainable intervention in EIS. As a community-based intervention it is empowering and there is potential benefit from developing nature connectedness, which is associated with increased conservation behaviours.^27^ Although further exploration of the benefits of green care, including its influence on psychotic experience and longer-term outcomes, is needed, the experiences this group describe suggest that nurturing opportunities for patients to access nature could promote recovery and rebalance relationships with the environment.

## Data Availability

The data that support the findings of this study are available on request from the corresponding author. The data are not publicly available owing to potential compromise of the privacy of those who contributed.

## About the authors

**Sharon Cuthbert** is a consultant psychiatrist with Sussex Partnership NHS Foundation Trust, based at Millview Hospital, Hove, UK. **Harriet Sharp** is an Academic Clinical Fellow in psychiatry with Sussex Partnership NHS Foundation Trust, Hove, UK. **Clio Berry** is a lecturer in Healthcare Evaluation and Improvement in Primary Care and Public Health at Brighton and Sussex Medical School, Brighton, UK.
